# Carbapenem resistance in a *Klebsiella quasipneumoniae* isolate resulting from production of CTX-M-33 and OmpK36 porin deficiency

**DOI:** 10.1128/aac.01214-25

**Published:** 2025-11-06

**Authors:** Jacqueline Findlay, Patrice Nordmann, Aurélie Jayol, Helena M. B. Seth-Smith, Adrian Egli, Laurent Poirel

**Affiliations:** 1 Medical and Molecular Microbiology, Faculty of Science and Medicine, University of Fribourg 98839, Fribourg, Switzerland; 2 Swiss National Reference Center for Emerging Antibiotic Resistance (NARA), University of Fribourg 27211, Fribourg, Switzerland; 3 Microbiology Unit, eHnv Hospital 240278 , Yverdon-les-Bains, Switzerland; 4 Institute of Medical Microbiology, University of Zürich 30990 , Zürich, Switzerland; Universita degli Studi di Roma "La Sapienza", Rome, Italy

**Keywords:** carbapenemase, CTX-M-33, OmpK36, *Klebsiella quasipneumoniae*, ESBL

## LETTER

Carbapenem-resistant *Klebsiella pneumoniae* (CRKP), particularly those producing carbapenemases, have continued to increase in incidence globally (1, 2), leading them to be listed as “critical” in the World Health Organization (WHO) Bacterial Priority Pathogen List (2). Therapeutic options for the treatment of infection caused by CRKP are severely limited, and as such, they are associated with higher mortality rates relative to their susceptible counterparts (3). Many countries now carry out routine surveillance of bacteria that produce carbapenemases. By comparison, extended-spectrum β-lactamase (ESBL)-producing gram-negative isolates receive less attention because ESBLs are considered endemic worldwide and, unlike carbapenemases, do not hydrolyze or confer resistance to “last-resort” carbapenem antibiotics (4). In addition, the novel ß-lactam-ß-lactamase inhibitor (BL/BLI) combination, cefepime-enmetazobactam (FEP-ENM), was recently approved by the European Medicines Agency for the treatment of infections caused by ESBL-producing gram-negative bacteria, offering an alternative and efficient therapeutic option besides carbapenems (5). Indeed, a recent study evaluating the activity of FEP-ENM on ESBL-producing *K. pneumoniae* reported that 98% of isolates exhibited susceptible MICs (≤4 mg/L) (6), suggesting that this combination is a valuable addition to the antibiotic armamentarium for the treatment of ESBL-producing *K. pneumoniae*. Here, we describe the case of a clinical *Klebsiella quasipneumoniae* isolate, recovered from a patient hospitalized in Switzerland, that exhibited resistance to the carbapenems and to FEP-ENM despite lacking any production of any ß-lactamase categorized as a carbapenemase.

In Spring 2025, a male patient in his 80s with a history of renal impairment and a kidney transplant 1 year prior, presented with acute graft pyelonephritis and was hospitalized. The patient had a history of *K. pneumoniae* infections, which had previously been successfully treated with imipenem. In this case he was treated empirically with imipenem for 3 days before being switched to ceftazidime-avibactam for 14 days following the isolation of a *K. pneumoniae* strain (VITEK MS identification) from the patient’s urine. Isolate N7039 showed resistance to carbapenems and was sent to the Swiss National Reference Center for Emerging Antibiotic Resistance (NARA) for further investigation. Antimicrobial susceptibility testing was performed by broth microdilution and interpreted according to the European Committee on Antimicrobial Susceptibility Testing (EUCAST) guidelines (7). We found that N7039 was resistant to all tested ß-lactams, including cephalosporins (ceftazidime, cefotaxime, and cefepime) and carbapenems (ertapenem, imipenem, and meropenem), according to the EUCAST guidelines. Additionally, it was resistant to the BL/BLI combinations ceftazidime-avibactam, aztreonam-avibactam (taking into consideration the breakpoint for aztreonam alone), imipenem-relebactam, meropenem-vaborbactam, and FEP-ENM, remaining susceptible to cefiderocol only (Table 1). Immunochromatographic tests targeting carbapenemase-encoding genes (NG-Test Carba 5, NG Biotech, Guipry, France) gave negative results, ruling out the production of a common carbapenemase. However, a positive signal was observed using the NitroSpeed Carba NP test (8), suggesting the production of a ß-lactamase able to hydrolyze carbapenems at least at a low level. PCR testing for ESBL-encoding ß-lactamase encoding genes (*bla*_CTX-M_ and *bla*_SHV_) gave a positive result for the *bla*_CTX-M_-encoding gene, and the sequencing of the corresponding amplicon (Microsynth AG) identified the *bla*_CTX-M-33_ gene. CTX-M-33 is a point mutant derivative of CTX-M-15 that has rarely been reported but was shown to exhibit low-level hydrolysis of meropenem (9). To allow for further genotypic characterization, the DNA from isolate N7039 was subject to both Illumina (short read) and Nanopore (long read) whole-genome sequencing (WGS). Hybrid assemblies were performed using UniCycler (10), and subsequent analyses were performed using the Center for Genomic Epidemiology Platform (https://www.genomicepidemiology.org/). Analysis of the WGS data allowed (i) a re-identification performed using Kmer Finder (11), which found that N7039 belonged to the *Klebsiella quasipneumoniae* species, with the latter being closely related to *K. pneumoniae* (cannot be separated with commercial databases on MALDI-TOF MS) (12) and (ii) an identification of the sequence type of that isolate, namely, ST2355 (Table 2). The *bla*_CTX-M-33_ gene was located onto a 138,495 bp IncR-type plasmid, on which the *bla*_SCO-1_ and *bla*_TEM-1_ genes encoding narrow-spectrum ß-lactamases were also identified (Table 2). The complete N7039 genome was deposited into GenBank under BioProject No. PRJNA1312282. Conjugation assays were performed to assess the mobility of this plasmid, named pN-CTX-M-33, using the sodium azide-resistant *Escherichia coli* J53 as a recipient strain as previously described (10), and its self-conjugative property at a frequency of 6.1 × 10^−7^ could be assessed.

**TABLE 1:** MICs of N7039, N7039 with complemented *ompK36*, recombinant strains expressing *bla*_CTX-M-33_, and the J53 transconjugant

	MIC (mg/L)/isolate						
Antibiotic	N7039	N7039/pACYC184	N7039/pACompK36	Top10	Top10/pTOPO-CTX-M-33	J53	J53/pN-CTX-M-33
Amoxicillin	>256	>256	>256	2	>256	2	>256
Amoxicillin/clavulanic acid	>256	>256	>256	2	>256	2	>256
Piperacillin	>256	>256	>256	2	>256	2	>256
Piperacillin/tazobactam	>256	>256	>256	2	>256	2	>256
Cefotaxime	>256	>256	>256	≤0.25	>256	≤0.25	>256
Ceftazidime	256	256	128	≤0.25	32	≤0.25	32
Ceftazidime/avibactam	16	16	2	≤0.25	0.5	≤0.25	0.5
Ceftazidime/enmetazobactam	256	256	4	≤0.25	0.5	≤0.25	0.5
Aztreonam	>256	>256	>256	≤0.25	>256	≤0.25	128
Aztreonam/avibactam	64	64	0.5	≤0.25	0.25	≤0.25	0.25
Aztreonam/enmetazobactam	>256	>256	2	≤0.25	0.25	≤0.25	0.25
Cefepime	>256	>256	>256	≤0.25	64	≤0.25	128
Cefepime/enmetazobactam	>256	>256	≤0.25	≤0.25	≤0.25	≤0.25	≤0.25
Ertapenem	>256	>256	1	≤0.015	0.25	≤0.015	0.06
Imipenem	16	16	0.125	0.06	0.125	0.06	0.125
Imipenem/relebactam	4	4	0.125	0.03	0.06	0.06	0.06
Imipenem/enmetazobactam	16	16	0.125	0.03	0.06	0.06	0.06
Meropenem	32	32	0.25	≤0.015	0.125	≤0.015	0.06
Meropenem/vaborbactam	32	32	≤0.015	≤0.015	0.03	≤0.015	≤0.015
Meropenem/enmetazobactam	32	32	≤0.015	≤0.015	0.03	≤0.015	≤0.015
Cefiderocol	1	1	1	≤0.06	0.25	≤0.06	0.25

**TABLE 2:** Genotypic characteristics of N7039 and plasmid pN-CTX-M-33^*a*^

			Resistance genes			Porins	
Isolate	ST	Size	Beta-lactamases	Others	Replicon types	OmpK35	OmpK36
N7039	2355	5,220,375 bp	*bla*_CTX-M-33_, *bla*_TEM-1_, *bla*_SCO-1_, *bla*_OKP-B-2_	*qnrB6*, *sul1*, *tetA*, *dfrA27*, *arr-3*, *oqxAB*, *fosA*, *aac(3)-IIa*, *aadA16*, *aac(6')-Ib-cr*	R	WT	Truncated - Y325stop
pN-CTX-M_33	NA	138,495 bp	*bla*_CTX-M-33_, *bla*_TEM-1_, *bla*_SCO-1_	*qnrB6*, *sul1*, *tetA*, *dfrA27*, *arr-3*, *aac(3)-IIa*, *aadA16*, *aac(6')-Ib-cr*	R	NA	NA

^*a*^ NA, not applicable.

The susceptibility testing of the CTX-M-33-producing recombinant *E. coli* TOP10 strain (9) and of the *E. coli* J53 transconjugant harboring pN-CTX-M-33 showed that CTX-M-33 conferred a small increase in the carbapenem MICs in accordance with what we previously reported (9) while causing a significant increase in MICs to the cephalosporins, aztreonam, and a small increase in the cefiderocol MIC. However, CTX-M-33 alone did not explain the elevated MICs to the carbapenems and BL/BLI combinations of isolate N7039.

The examination of the DNA sequences of the genes encoding the outer membrane porins OmpK35 and OmpK36 identified that while N7039 harbored a wild-type *ompK35*, *ompK36* was truncated (Y325stop). OmpK36 is known to be the primary route by which carbapenems enter the *K. pneumoniae* and *K. quasipneumoniae* cells, and its loss has been linked to elevated carbapenem MICs (13). Hence, it is likely that the absence of a functional OmpK36 played a role in the elevated carbapenem MICs and BL/BLIs, which encompassed a carbapenem observed in N7039 (Table 1). To test this hypothesis, a wild-type *ompK36* gene was cloned and expressed in the low-copy number plasmid pACYC184, pACompK36, and transformed into clinical isolate N7039 by electroporation. Subsequent MIC testing showed that MICs of the carbapenems and their respective BL/BLI combinations were significantly reduced in the *ompK36* complemented strains (ETP, >256-fold, IPM; 64-fold, MEM; 128-fold reductions), confirming the impact of OmpK36 loss in N7039 (Table 1). Interestingly, the MICs of FEP-ENM but not of FEP were reduced from >256 to ≤0.25 mg/L, strongly suggesting that a functional OmpK36 is critical for the entry of ENM into the bacterial cell and ultimately identifying a major shortcoming of this novel drug combination.

Our study indicates that in the absence of carbapenemase, or at least of a ß-lactamase being so far categorized as a carbapenemase considering the weak carbapenemase activity of CTX-M-33, simply the loss of porin OmpK36 combined with the production of CTX-M-33 might be sufficient to confer a relatively high-level of carbapenem resistance in *K. quasipneumoniae*. Such impact of an ESBL-possessing weak carbapenemase properties has previously been highlighted with some GES derivatives (14).

The impact of OmpK36 loss on the activity of FEP-ENM was also identified and may be an important factor to consider when using this BL/BLI combination and requires further investigation. Finally, we report here the emergence of CTX-M-33 in Switzerland, which can be considered a worrying ESBL-type enzyme. Although the original report of CTX-M-33 production corresponded to a *K. pneumoniae* ST405 isolate, recovered in Portugal, exhibiting reduced susceptibility to carbapenems and harboring *bla*_CTX-M-33_ on an IncFIB-type plasmid (9), the present report highlights its occurrence in a *K. quasipneumoniae* isolate mediated by a different plasmid type.
